# Xylanase Supplementation in Wheat-Based Diets of Laying Hens Affects the Egg Yolk Color, Carotenoid and Fatty Acid Profiles

**DOI:** 10.3390/foods11152209

**Published:** 2022-07-25

**Authors:** Georgios A. Papadopoulos, Styliani Lioliopoulou, Stella A. Ordoudi, Ilias Giannenas, Veerle Van Hoeck, Dany Morisset, Georgios Arsenos, Paschalis Fortomaris, Fani T. Mantzouridou

**Affiliations:** 1 Laboratory of Animal Husbandry, Faculty of Veterinary Medicine, Aristotle University of Thessaloniki, 54124 Thessaloniki, Greece; slioliopo@vet.auth.gr (S.L.); arsenosg@vet.auth.gr (G.A.); fortomap@vet.auth.gr (P.F.); 2 Laboratory of Food Chemistry and Technology, School of Chemistry, Aristotle University of Thessaloniki, 54124 Thessaloniki, Greece; steord@chem.auth.gr (S.A.O.); fmantz@chem.auth.gr (F.T.M.); 3 Laboratory of Nutrition, Faculty of Veterinary Medicine, Aristotle University of Thessaloniki, 54124 Thessaloniki, Greece; igiannenas@vet.auth.gr; 4 Kemin Europa N.V., Animal Nutrition and Health EMENA, Toekomstlaan 42, 2200 Herentals, Belgium; veerle.vanhoeck@kemin.com (V.V.H.); dany.morisset@kemin.com (D.M.)

**Keywords:** eggs, laying hens, carotenoids, wheat, fatty acids

## Abstract

Wheat is rich in non-starch polysaccharides (NSP) and their degradation in poultry diets is promoted by exogenous carbohydrases. The objective here was to evaluate the effect of adding an intrinsically thermostable xylanase on wheat-based diets for laying hens in yolk color, carotenoid and fatty acid profiles of eggs. A total of 128 laying hens were used for 12 weeks. They were randomly allocated to four dietary treatments with different levels of xylanase: T1: control (no xylanase), T2: 30,000 U/g, T3: 45,000 U/g and T4: 90,000 U/g, with 32 birds, 16 replicates per treatment (2 birds/replicate). At the end of the experimental period, egg yolk color index, redness (a*) and yellowness (b*) of egg yolks were found significantly higher in all the enzyme supplemented diet groups (T2, T3, T4) compared with the control (T1). Canthaxanthin levels were significantly higher in T3 than T1 (*p* < 0.05). Total n-3, n-6 and total polyunsaturated fatty acids (FAs) were significantly higher in T4 compared with the control (*p* < 0.01), while the reverse trend was evidenced for monounsaturated FAs. Additionally, total n-3 FAs were higher in the T2 than T1 (*p* < 0.005). Overall, the results showed that exogenous xylanase enzyme supplementation in wheat-based diets for laying hens contribute to maintaining egg yolk color. Overall, exogenous xylanase enzyme supplemented at all levels in wheat-based laying hens’ diets improved egg yolk color compared to the control diet. The enzyme supplemented at the higher level (90,000 U/g) improved polyunsaturated and reduced monounsaturated egg yolk fatty acid content.

## 1. Introduction

Enzymes have been widely used as feed additives in animal production, in order to achieve better digestibility, nutrient utilization and costs reduction [1]. This is of special concern for monogastric animals (pigs and poultry) that lack the appropriate enzymes to break down non-starch polysaccharides (NSP) such as hemicelluloses and xylan in animal feed [2,3]. Xylanases, a group of microbially produced hydrolytic enzymes are capable to break down xylan into xylose [3], resulting in reduction of gut viscosity, release of encapsulated nutrients and increase in dietary energy available [4,5]. So far, studies with dietary supplementation of xylanase in laying hens have shown improved productivity parameters and nutrient utilization [2,6,7,8]. More specifically, a study conducted by Bobeck et al. [7] showed that the beneficial effects of xylanase supplementation in layers’ diets include increased egg production, egg mass and feed intake. These results come in agreement with the results of another study [9], which showed that xylanase supplementation in layers’ wheat-rich diets results in improved performance and production traits.

The beneficial effects of dietary xylanase supplementation of laying hens depend on the diet composition and energy levels, as these parameters play a key role on the enzyme activity. Wheat-based diets contain high amounts of NSP, so xylanase supplementation is an important cost-saving strategy, as it reduces the anti-nutritive effect of NSP. Moreover, enzyme preparations containing mixtures of different types of hydrolytic enzymes may expand the beneficial effects to more animal feed types. A study in laying hens [10] showed that supplementation of both xylanase and β-glycanase in maize/soybean meal-based diets improved feed conversion ratio. The relevant research so far focused mainly on growth performance of birds, while the available literature about how xylanase affects egg quality parameters is still very limited. The new xylanase product Xygest HT has been studied in broilers and was found to be beneficial for performance parameters such as growth performance, organ weights, intestinal viscosity and pH [11]. Recently, Nguyen et al. (2021) [12] showed an improvement on egg yolk color following xylanase supplementation in laying hens fed wheat-based diets. Nevertheless, the latter study did not evaluate further egg yolk carotenoid and fatty acid content. In the case of increased viscosity due to high NSP dietary content, the effect of nutrient absorption will be affected. Usually, the diets used for laying hens are corn-soybean-based and their carotenoid content is low and therefore, exogenous pigments, such as lutein and canthaxanthin, are supplemented to the diet to improve yolk color [13]. Thus far, the research data on the effects of NSP enzymes in laying hens fed wheat-based diets on egg yolk carotenoid and fatty acid content is limited. It is known that carotenoids are absorbed together with dietary lipids by forming mixed micelles [14]. It is plausible that an improvement in nutrient digestion and absorption by xylanase supplementation could be reflected in carotenoid and fatty acid content of egg yolks.

Previous studies have reported the effects of supplementing exogenous enzymes on laying hens’ diets on egg yolk fatty acid levels. Abd El-Hack et al. [15] found no significant effects on egg yolk lipid profile in diets containing different inclusion levels of corn distiller’s dried grains with solubles (DDGS) and supplemented with a multi-enzyme combination of xylanase, protease and amylase in laying hens’ diets. Similar effects were reported in a later study by [16], in which supplementation of an enzyme mixture did not result in an altered egg lipid profile in laying hens fed variable levels of DDGS. Elsewhere, xylanase supplementation of laying hen diets containing DDGS, wheat and corn did not result in a significant effect in yolk lipid profile [17]. In corn-soybean-based diets supplemented with flaxseed, xylanase addition at several levels led to variable effects in egg yolk lipid content [18]. Specifically, xylanase supplementation led to a reduction of linoleic acid and arachidonic acid content, and increased alpha-linolenic acid content [18]. Enzyme supplementation did not affect the levels of long chain n-3 fatty acids, while overall total n-3 fatty acids were higher in the group supplemented with the greater level of enzyme compared to the non-supplemented diet [18]. In the study of Jia et al. [19], multicarbohydrase enzyme supplementation resulted in an increase of n-3 and docosahexaenoic acid content in eggs from hens fed the flaxseed diet or a mixture of flaxseed and peas compared to a canola seed supplemented diet. The latter authors postulated that this effect may be due to the depolymerization of the cell wall polysaccharides by enzyme action, which increased fat availability in the gut and for the digestive enzymes to act.

It was hypothesized that based on the properties of xylanase NSP enzyme, the digestibility and the absorption of specific dietary nutrients such as carotenoids and fatty acids would be improved in wheat-based diets from the xylanase, supplemented group. The objective of the present study was to evaluate the efficacy of the new thermostable xylanase produced by *Komagataella phaffi* yeast in wheat-based laying hens’ diets, when supplemented at 30,000 U/g (10 g/t), 45,000 U/g (15 g/t), and 90,000 U/g (30 g/t) compared to a control non-supplemented diet with enzyme on egg yolk color, carotenoid and fatty acid profile.

## 2. Materials and Methods

### 2.1. Ethical Considerations

Experimental procedures were approved by the Ethical Committee branch of the Research Committee of Aristotle University of Thessaloniki, Greece (approval number 71071/2020). The animal phase of the experiment was designed considering all welfare requirements described by Good Farming Practice Guidelines [20].

### 2.2. Experimental Design

The xylanase product was Xygest^TM^ HT (Kemin Animal Nutrition and Health, Herentals, Belgium). This is an intrinsically thermostable monocomponent xylanase produced by *Komagataella phaffi* and is a beta 1–4,endo-xylanase enzyme belonging to the GH11 family, designed to improve the degradation of dietary fiber to maximize energy utilization of the diet. Xygest HT has a minimal xylanase activity of 3,000,000 U/g on a corn starch-based carrier.

The study took place in a designated poultry house located in Galatista, municipality of Chalkidiki of Greece. Laying hens were housed in experimental cages and each cage had dimensions of 41 cm × 41 cm, which ensured more space per hen (840.5 cm^2^) than the minimum requirements of recent EU directive (at least 750 cm^2^ of cage area per hen) [21].

In total, 128 laying hens (Isa Brown breed) were transferred in the research facility at the age of approximately 18 weeks. One week later (week 19) all hens were weighed and allocated in pairs in individual cages to cater to the needs of experimental replicates. There were 16 replicates per treatment (64 cages in total) with each replicate containing two hens. Laying hens were fed the experimental diets since their 19th week of age which was considered the adaptation week (AW1). Diets were formulated in accordance with ISA Brown commercial product guide [22]. The main ingredients and calculated nutrient analysis of the diets are shown in Table 1. The feed was in meal form and not pelleted. The enzyme quantities were added during the mixing procedure together with the premix of minerals and vitamins. The experimental period started when hens reached about 50% and more of their laying capacity, which occurred at the age of 20 weeks. The actual duration of the experimental period was 12 weeks. Temperature was kept within the range between 20–24 °C during the experimental period, and relative humidity between 55–70%. Light period was within the recommended range of 14 to 16 h of light duration based on ISA brown management guide [22].

Feed samples were analyzed at the ILVO institute in Belgium (sample code = 2020/1576/99557| analysis date = 13 July 2020). Initially, feed samples were ground through a 1 mm screen (Wiley, Rheotec, Maarkedal, Belgium). The chemical composition of dry feed was characterized by analyzing moisture, crude protein, crude fat, crude ash, NDF (neutral detergent fiber), starch and sugars as previously described by De Boever et al. [23]. More specifically, residual moisture was determined by drying at 103 °C [24]. Crude ash was obtained by incineration at 550 °C [25]. The CP (N × 6.25) was determined according to Kjeldahl [26]. The crude fat was extracted with petroleum-ether after hydrolysis with HCl [27]. Crude fiber was obtained with the Ankom Fiber Analyzer (Ankom Technology, Macedon, NY, USA) after boiling subsequently with sulfuric acid and sodium hydroxide [28]. NDF was determined with the Ankom Fiber Analyzer using α-amylase and sodium sulfite and expressed on ash-free basis [29]. The DDGS samples were more extensively analyzed. ADF (acid detergent fiber) was determined with the Ankom Fiber Analyzer and expressed exclusive ash; the residue was then treated with sulfuric acid to obtain ADL (acid detergent lignin) [29]. By N-analysis of the AD-residue, acid detergent insoluble CP (ADICP) was obtained. Starch (STA) was determined after autoclaving and hydrolysis with amyloglucosidase [30]. Sugars (SUG) were extracted with 40% ethanol and analyzed according to the Luff Schoorl method [31]. Diets were also characterized by the following approach: NSP = 100—moisture—crude protein—crude fat–crude ash—starch—sugar. Then, as the NSP are a heterogenic fraction, composed from cell membranes and pectins, these two fractions were separated by determination of NDF. NDF is a factor for all cell membrane fractions. The NDF-fraction concerns the total cell-wall content, which we can further split up in hemicellulose, cellulose and lignin. Hemicellulose is calculated as NDF—ADF and cellulose as ADF—ADL. Then, by doing NSP—NDF, an indication of the pectins can be obtained. Further, the fraction of residual non-starch polysaccharides (RNSP) as a measure of pectins, xylans, arabans and beta-glucans was measured by difference: 100—moisture—crude protein—crude fat—crude ash—NDF—starch—sugars. Results of the analysis are shown in Table 2.

#### Enzyme Activity Recovery in Feed Samples

Before the start of the trial, feed samples from each treatment were provided to Kemin Europa N.V. for recovery analysis of the xylanase. The xylanase activity of a feed extract was determined using the Xylazyme AX tablet test. Xylazyme AX tablets, containing dye-labeled cross-linked wheat xylan polymer as a substrate for enzymatic action, were suspended in the extract. The xylan was hydrolyzed by the xylanase present in the extract, liberating color into the solution. The xylanase activity in the extract was quantified by measuring the absorbance of the solution and comparing with absorbances, obtained for the standard curve. The dosages and enzyme activities used in the present study are presented in Table 3.

### 2.3. Chemicals and Reagents

All chemicals and reagents used in this study were of analytical or HPLC grade. Butylated hydroxytoluene (BHT) (≥99%) was purchased from Sigma-Aldrich and sodium sulfate were provided by Panreac Quimica. Solvents used, namely, acetone, methyl-tert-butyl ether (MTBE), methanol and water, were all purchased from Sigma Co. (St. Louis, MO, USA). Lutein (≥88%) was from Glentham (Corsham, United Kingdom) and zeaxanthin (≥95%) from Carbosynth (Bratislava, Slovakia); all-*trans*-Canthaxanthin (≥86%) analytical standard was obtained from LGC standard (Wesel, Germany). Pentadecanoic acid, chloroform, BHT, methanolic sodium methoxide, pure methyl esters were purchased from Sigma Co. (St. Louis, MO, USA). Boron trifluoride and methanol were obtained from Fluka (Buchs, Switzerland).

### 2.4. Egg Yolk Color Evaluation

At the start of the experiment (completion of week 1) and at 4, 8 and 12 weeks of the experimental period, egg quality parameters were measured on sixteen (16) randomly selected eggs per treatment (one from each replicate/pen) and per sampling period. Hence a total of *n* = 256 eggs were collected and analyzed individually.

Yolk color was scored visually by using the Yolk Color Fan^®^ scale and also measured instrumentally with Chroma meter CR-410 (Konica Minolta, Osaka, Japan) using the L*a*b* color space. The L* value represents lightness (0 = black, 100 = white), a* indicates redness (−100 = green, 100 = red) and b* gives a value for yellowness (−100 = blue, 100 = yellow) [32]. For technical reasons, the Chroma meter CR-410 was not used for eggs collected at the start of the experiment at 20 weeks of age (baseline measurement), but was used for the rest of the measurements (at 4th, 8th and 12th week of the experimental period).

### 2.5. Egg Yolk Carotenoid Profile and Content

#### 2.5.1. Carotenoid-Rich Extract Preparation

In total, 40 out of the 256 egg yolks (*n* = 10 per each experimental group, which were collected at the 8th experimental week) were randomly collected and stored in individual containers at −20 °C. The frozen samples were lyophilized in a Christ Alpha 1–2 freeze dryer (Osterode, Germany), sealed at a Besser Chamber Vacuum (Dignano, Italy) machine and stored at 4 °C until further analyses.

Extracts were prepared according to Papadopoulos et al. (2019) [33] with slight modifications, as follows: a portion of 0.5 g of the freeze-dried, homogenized yolk powder was placed directly into a polystyrene falcon tube (40 mL). The sample was extracted with 10 mL of ice-cold acetone solution containing butylated hydroxytoluene (BHT) (0.05% *w*/*v*), with the aid of an ultrasonic probe (Bandelin Sonoplus HD 2070, Berlin, Germany) (70 W, 97% amplitude) and for a total of 3 min to achieve complete discoloration of the yolk powder (1 cycle). The acetone extracts were centrifuged at 10,000× *g*, 4 °C, for 20 min. The extraction was carried out in duplicate. The sample temperature did not exceed 20 °C with the aid of an ice bath. All procedures were performed under dim light conditions.

#### 2.5.2. Total Carotenoid Content Estimation

Each supernatant was diluted with pure acetone into a 10 mL volumetric flask. Visible absorbance spectra within 340–700 nm were acquired using a Shimadzu UV-1601 spectrophotometer (Kyoto, Japan). The total carotenoid content of each sample was assessed after reading the absorbance values at 450 nm and using an extinction coefficient of 2340. Results were expressed as zeaxanthin equivalents (μg of zeaxanthin/g egg yolk on dry basis). The supernatant solutions were pooled and the solvent was evaporated until constant weight using a Buchi Rotavapor R-100 (Flawil, Switzerland). The residue was stored at −20 °C until HPLC analyses. Prior to injection, the sample was dissolved in 2 mL of a mixture of MeOH/MTBE/H_2_O (45.5:52.5:2 *v*/*v*/*v*), vortexed and filtered through a 0.45 µm pore membrane filter.

#### 2.5.3. RP-HPLC-DAD Analyses of Individual Carotenoids

Individual carotenoids were analyzed using a Shimadzu Nexera X2 UHPLC System (Shimadzu Corporation, Kyoto, Japan), equipped with a LC-30AD pump, a SIL-30AC autosampler (50 μL loop), a CTO-20AC column oven, and a UV-Visible diode array SPD-M30A detector (DAD). Separation was achieved on a C30 reverse phase column (YMC-Pack YMC C30, 250 mm × 4.6 mm id, S-5 μm, YMC Co., Ltd., Kyoto, Japan), under elution conditions described previously [34]. Briefly, a linear gradient change in eluent composition from 100% A (MeOH/MTBE/H_2_O, 81:14:4, *v*/*v*/*v*) to 50% B (MeOH/MTBE, 10:90, *v*/*v*) was achieved within 30 min, and then back to the initial conditions in 10 min. The flow rate was set at 1 mL/min, the column temperature at 32 °C, and the injection chamber at 4 °C. The injection volume was 5 μL for extracts and 10 μL for standard compounds. Data acquisition and analysis were carried out using Lab Solution ver. 5.86 software (Shimadzu Corporation, Kyoto, Japan). All analyses were performed in duplicate.

Individual carotenoids were tentatively identified on the basis of UV-Vis spectral characteristics (λ_max_), retention time, and elution order on C30 column in comparison with those of available standard compounds. External standard calibration curves were prepared using stock solutions of zeaxanthin and canthaxanthin in acetone (containing BHT 0.05%) and hexane-THF, respectively, after appropriate dilutions with the sample solvent. The concentration of stock solutions was calculated spectrophotometrically with the aid of the following equation:

C (mg/L) = 10,000 × DF × A_λmax_/E^1%^ where, DF, the dilution factor, λmax the wavelength of maximum absorbance (450 nm for zeaxanthin and 470 nm for canthaxanthin), E^1%^ the extinction coefficient values (2340 for zeaxanthin in acetone and 2200 for canthaxanthin in hexane). Linear five-point calibration curves were constructed by plotting the respective analyte peak area against the injected mass that was corrected for purity and ranged between 2–40 ng for all-*trans*-zeaxanthin and 5–60 ng for all-*trans*-canthaxanthin (R^2^ > 0.993); all-*trans*-Lutein concentration was calculated using the zeaxanthin calibration curve.

### 2.6. Analysis of Fatty Acid Profile and Content in Egg Yolk

The fatty acid profile in egg yolk was analyzed using Gas Chromatography coupled with Flame Ionization Detector (GC-FID). Specifically, 10 μL of 200 mg/mL pentadecanoic acid (Sigma, St. Louis, MO, USA) in chloroform as internal standard were mixed thoroughly with approximately 50.0 ± 0.1 mg of egg yolk in a test tube before extracting lipids according to Dole [35]. Extraction began by adding 950 µL of lipid extraction solvent (chloroform-methanol 2:1 (*v*/*v*) with BHT (0.005% *w*/*v*) (Sigma, St. Louis, MO, USA). After 5 min, 200 µL of water were added, and the mixture was stirred vigorously for 1 min to afford extraction of the lipids. After centrifugation for 2 min at 1500× *g*, the underlying layer was removed, condensed under a stream of nitrogen, and fatty acid methyl esters (FAMEs) were prepared by addition of 0.5 mL boron trifluoride in methanol (Fluka, Buchs, Switzerland), incubation at 50 °C for 10 min, addition of 0.5 mL of methanolic sodium methoxide (Sigma), and incubation at 50 °C for another 10 min. FAMEs were extracted by adding 1 mL of hexane, stirring vigorously for 1 min, and analyzing the supernatant in an Agilent 7890A gas chromatograph (Waldbronn, Germany) equipped with ultra-inert inlet liner, an Agilent HP-88 column (length 100 m, internal diameter 0.25 mm, film thickness 0.20 μm), and flame ionization detector. The column temperature was programmed from 120 °C, held for 1 min, to 175 °C at 10 °C/min, held for 10 min, from 175 °C to 210 °C at 5 °C/min, held for 5 min, and from 210 °C to 230 °C at 5 °C/min, held for 6 min. The carrier gas was helium at a constant flow of 2 mL/min and the split ratio was 1:50. Methyl esters of individual fatty acids were identified in the chromatograms by comparing their retention times to those of standard compounds and were quantified by comparing the area under their peaks to that of methyl pentadecanoate (derived from the internal standard) using the Agilent ChemStation software (Santa Clara, CA, USA).

Additionally, the Atherogenic (AI) and Thrombogenic (TI) indices and the ratio between hypocholesterolemic and hypercholesterolemic fatty acids (h/H) were calculated using the following formulas [36,37]:

(i) AI = (4 × C14:0 + C16:0 + C18:0)/(ΣMUFA + ΣPUFA-n-6 + ΣPUFA-n-3)
(ii) TI = (C14:0 + C16:0 + C18:0)/(0.5 × ΣMUFA + 0.5 × ΣPUFA-n-6 + 3 × ΣPUFA-n-3 + ΣPUFA-n-3/ΣPUFA-n-6)
(iii) h/H = C18:1n9c + C18:2n6c + C18:3n3c + C18:3n6c + C20:2n6 + C20:3n6 + C20:4n6 + C22:6n3/C14:0 + C16:0

### 2.7. Statistical Analysis

The pen (replicate) was the experimental unit. Each treatment had 2 hens per replicate and performance parameters were evaluated per pen (replicate). The effect of dietary treatments on the parameters investigated in the present study was analyzed with one-way ANOVA using SPSS 25.0 (IBM SPSS Statistics for Windows, Version 25.0. Armonk, NY, USA: IBM Corp.). The dietary treatment groups (T1, T2, T3, T4) were included as fixed factors in the statistical model. Post hoc comparisons between treatments were investigated by Tukey’s test. The average values including the standard deviation of the mean were calculated for every examined parameter. Furthermore, statistical evaluation of carotenoids and of major fatty acid categories were carried out by using ANOVA and multiple comparisons between treatment means (Tukey’s test) with the use of GraphPad Prism (version 9.1.2 for Windows^®^, GraphPad Software, San Diego, CA, USA). The level of significance for all statistical evaluations was set at *p* < 0.05.

## 3. Results

### 3.1. Egg Yolk Color Evaluation

**Figure 1:**
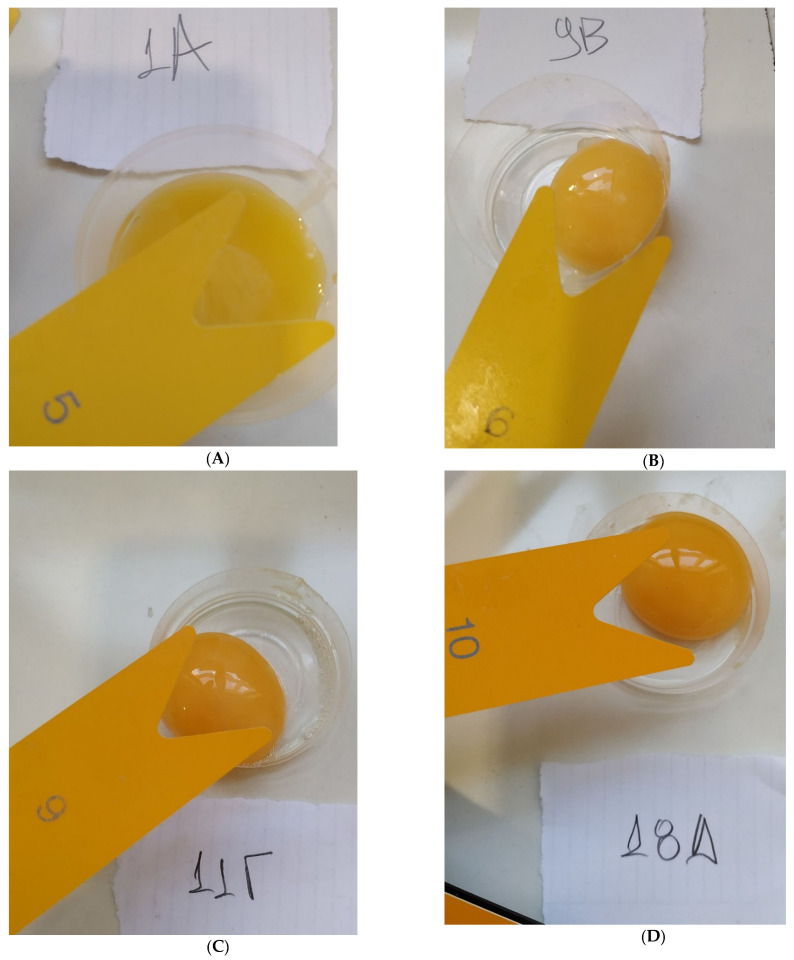
Representative images of egg yolks showing the respective Yolk Color Fan^®^ index of eggs collected at the 8th week of the experimental period: (**A**) T1: control-no enzyme; (**B**) T2: 30,000 U/kg; (**C**) T3: 45,000 U/kg; (**D**) T4: 90,000 U/kg. The Greek letters were initially used to encode the dietary treatments, while each number corresponds to one individual hen.

As aforementioned, egg quality parameters, including egg yolk color, were measured at the end of 1st, 4th, 8th and 12th week of the experiment. The results are shown in Table 4 and Table 5. In Figure 1, representative images of egg yolks and the respective Yolk Color Fan^®^ index of eggs collected at the 8th week of the experimental period are shown.

At the end of the 1st experimental week, yolk color visual measurements showed significant differences (*p* < 0.001) between the four treatments, with T1 showing the lowest values and T3 and T4 the highest for the overall period.

Later on, at the end of the 4th experimental week, Lightness (L*) and redness (a*) of the yolk color were found significantly more intense in T4 group than in the other groups. Yellowness (b*) was increased in T4 than T2 (*p* = 0.020). Gradually, the effect of xylanase supplementation to the egg yolk color became evident with lower dose; thus, at the end of the 8th experimental period, yolk color (redness and yellowness) remained darker in T3 and T4 than in the control group (*p* < 0.001). Redness (a*) was higher in all groups supplemented with the enzyme at different doses (T2, T3, T4) than the control non-supplemented group (T1) (*p* < 0.001). Yellowness (b*) was higher in the T3 and T4 groups than in T1 (*p* = 0.017). Similar observations were made also at the end of the 12th week; yolk color index was higher in the T2, T3 and T4 group compared to T1 (*p* < 0.001). Lightness (L*) was greater in the T1 group compared to T2 (*p* = 0.038). Redness (a*) was higher in T3 and T4 groups than T1 (*p* < 0.001).

The overall experimental period data analysis showed that yolk color measurements were significantly affected by the xylanase dose treatment. Specifically, yolk color index and redness (a*) was significantly higher in all supplemented with the enzyme groups (T2, T3, T4) compared to the control group (T1) (*p* < 0.001). Yellowness (b*) was the highest in T4 group than in T1 (*p* = 0.014).

### 3.2. Egg yolk Carotenoid Content and Profile

**Figure 2:**
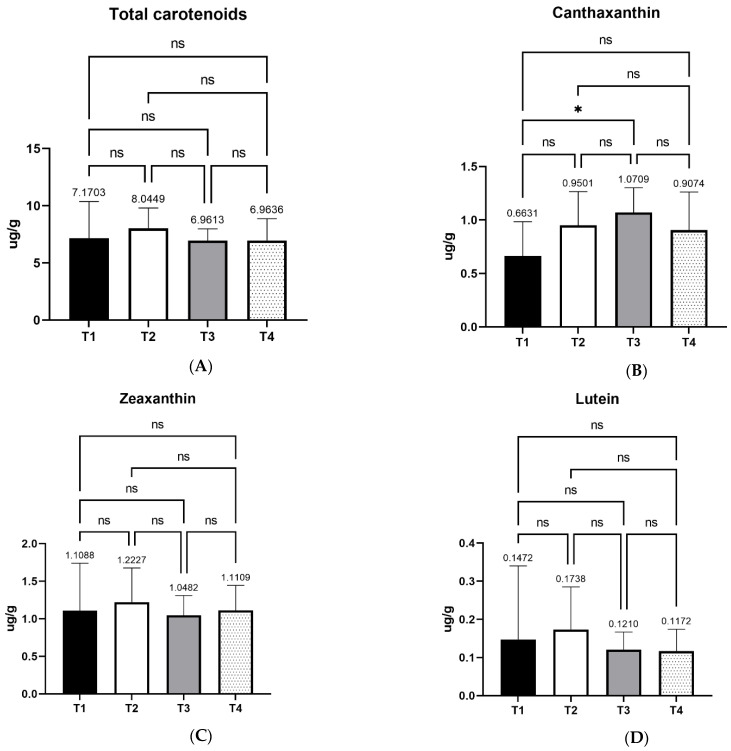
Effects of four xylanase dietary treatments (T1–T4) of laying hens on egg yolk carotenoid content, expressed as ug/g on dry basis (mean ± SD): Panel (**A**) represents the total carotenoid content of egg yolk; panel (**B**) the Canthaxanthin content; panel (**C**) the zeaxanthin content, and panel (**D**) the lutein content, all expressed as ug/g of egg yolk. T1: control-no enzyme; T2: 30,000 U/kg; T3: 45,000 U/kg; T4: 90,000 U/kg. *: mean values differ significantly (*p* < 0.05). ns: not significant.

The results of treatment effects on egg yolk carotenoid content and profiles are shown in Figure 2. The photometrically assessed total carotenoid contents were not found to differ significantly between the four treatments (*p* > 0.05). The same was observed also for individual carotenoids, mainly all-*trans* forms of lutein and zeaxanthin. On the other hand, the total content of canthaxanthin analogues increased significantly (*p* < 0.05) upon the treatment with xylanase. Their content was higher in T3 treatment compared with T1 (control). This result implies that the xylanase treatment facilitates the absorption, transport, and deposition of canthaxanthin over the other types of xanthophylls in the egg yolk of the wheat-based-fed laying hens.

### 3.3. Egg Yolk Fatty Acid Content

Egg yolk fatty acid profile obtained by fatty acid analysis is shown in Table 6. ANOVA analysis showed that there are significant differences (*p* < 0.05) in certain fatty acids between the four dose treatment groups.

**Figure 3:**
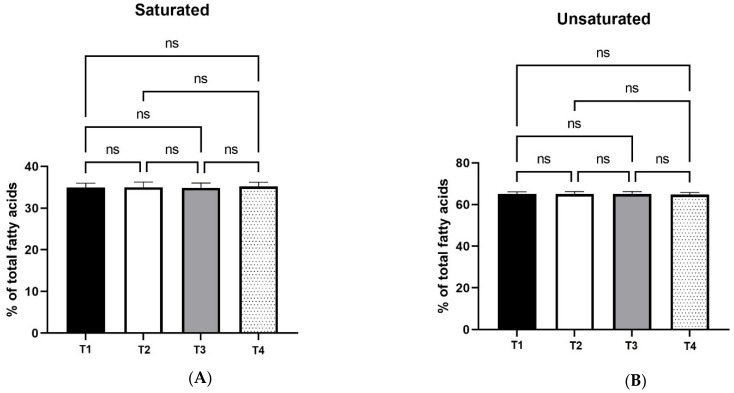

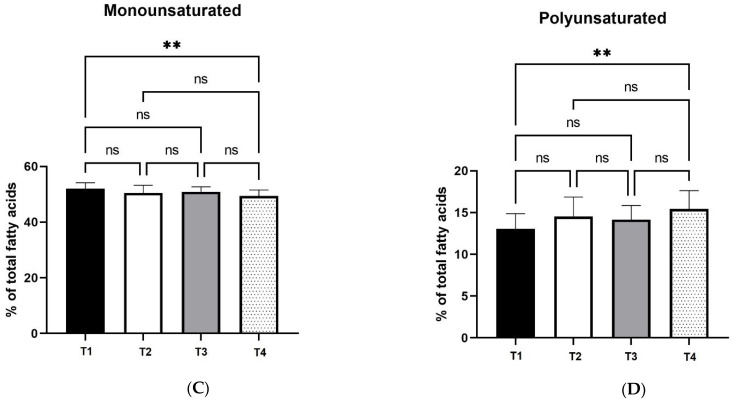
Effects of four xylanase dietary treatments (T1–T4) of laying hens on saturated (panel **A**); unsaturated (panel **B**); monounsaturated (panel **C**), and polyunsaturated (panel **D**) fatty acid percentage of egg yolks (mean ± SD). T1: control-no enzyme; T2: 30,000 U/kg; T3: 45,000 U/kg; T4: 90,000 U/kg. **: mean values differ significantly between them (*p* < 0.01); ns: not significant.

Moreover, the statistical analysis of fatty acid profile data showed no significant differences in saturated and unsaturated fatty acid percentages between the four treatments, as shown in Figure 3.

In comparison with the control (T1), the highest xylanase dosage treatment (T4) of the laying hen diet induced significant changes (*p* < 0.01) in the % contents of total monounsaturated (MUFA) and total polyunsaturated fatty acids (PUFA) in the egg yolk. More specifically, T4 treatment resulted in reduced monounsaturated fatty acid levels and increased polyunsaturated fatty acid levels in comparison with T1 (control) treatment. Figure 3 graphically represents these results.

**Figure 4:**
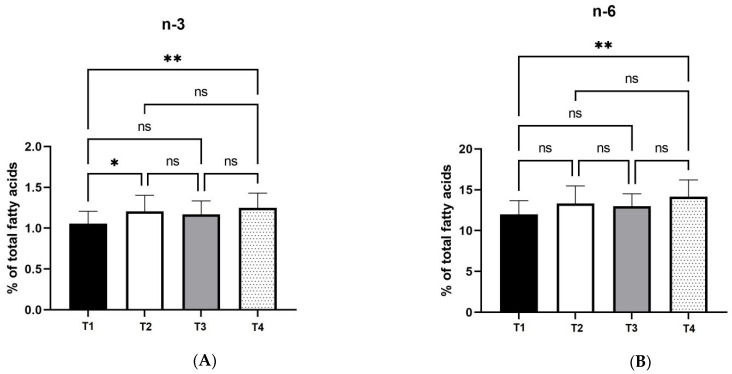

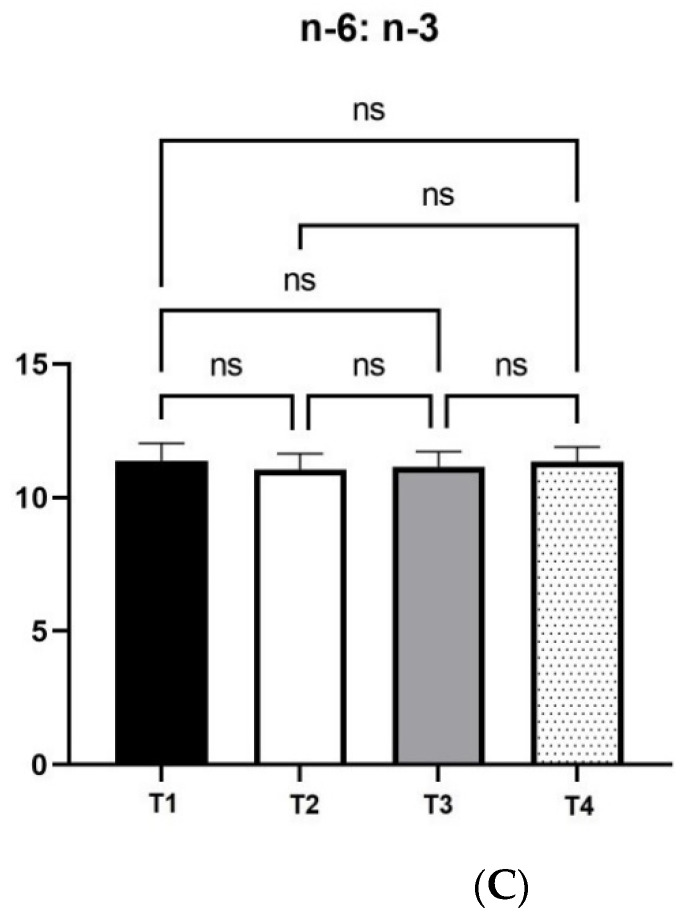
Effects of four xylanase dietary treatments (T1-T4) of laying hens on n-3 (Panel **A**); n-6 (Panel **B**), and n-6: n-3 ratio (Panel **C**) of egg yolks (mean ± SD). T1: control-no enzyme; T2: 30,000 U/kg; T3: 45,000 U/kg; T4: 90,000 U/kg. *: mean values differ significantly between them (*p* < 0.05); **: mean values differ significantly between them (*p* < 0.01); ns: not significant.

It should be stressed though that, the n-6: n-3 ratio did not change significantly with enzyme treatments (Figure 4C). There was also a significant (*p* < 0.05) increase in n-3 egg yolk content in T2 treatment compared with T1 (control). These results are shown in Figure 4.

**Figure 5:**
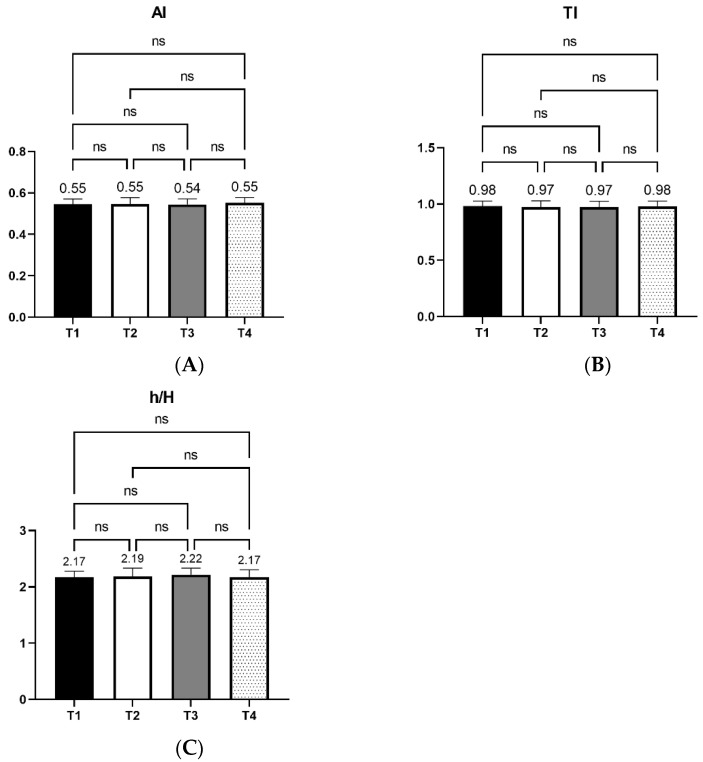
Effects of four xylanase dietary treatments (T1–T4) of laying hens on Atherogenic (AI, Panel **A**) and Thrombogenic (TI, Panel **B**) indexes, and the ratio between hypocholesterolemic and hypercholesterolemic fatty acids (h/H, Panel **C**) of egg yolks (mean ± SD). T1: control-no enzyme; T2: 30,000 U/kg; T3: 45,000 U/kg; T4: 90,000 U/kg. ns: not significant.

In Figure 5, the results regarding the AI, TI, and h/H indexes are presented. According to the results, there was no significant difference between treatments.

## 4. Discussion

Consumer requirements and preferences on animal products are particularly important for the animal product industry. The nutrition value and the organoleptic characteristics of eggs are the major parameters by which consumers judge the quality of eggs. In this study, three xylanase supplementation levels were used and their long-term effects on egg quality were evaluated. Laying hens and broilers are fed mainly with corn-based diets worldwide, so it is important to find alternative feed ingredients available for the poultry industry with lower cost and without negative effects on egg quality parameters [38,39,40,41]. The findings of this study, which used a wheat-based diet with xylanase supplementation, are promising regarding this direction.

More specifically, although consumer preferences about egg yolk color differ between countries, culture and traditions, in Europe, the consumers prefer egg yolks with darker hue [42]. Corn-based diets that are naturally rich in carotenoids offer this advantage and result in egg yolks with a better color score. However, the main carotenoid found in corn is lutein, which increases the yellowness but does not enhances the yolk’s red pigmentation properly [43]. In this study, significant findings were observed for the egg yolk coloring parameters. Specifically, it was revealed that enzyme supplementation (at all levels used) in a wheat-based diet improved egg yolk color significantly compared to the control group. A previous study [44] showed no significant effects on egg yolk pigmentation after dietary supplementation of a cellulose and xylanase mixture in laying hens’ alfalfa and rye diets. Another study showed improvement of egg yolk color in hens feeding wheat-triticale diet supplemented by an enzyme complex containing xylanase, glucanase, hemicellulose and amylase [45]. A recent study [12] showed that the supplementation of laying hens with 12,000 BXU of xylanase per kg of NSP-rich feed improved egg yolk color score. The researchers measured egg yolk coloration only visually, using the Roche scale. In our study, the approach of measuring egg yolk color included visual and instrumental methods, while spectrophotometric/chromatographic analysis was used for carotenoids, the main pigments that determine egg yolk coloration. The beneficial effect of xylanase in egg yolk coloration is a finding of practical importance for the feed industry targeting laying hen enterprises. Formulating diets with increasing wheat quantities and decreasing of corn may raise concerns about egg yolk coloring, since carotenoid content of wheat is extremely low compared to that of corn. The present study shows that by using the xylanase enzyme product, even at the lower supplementation level, these negative effects could be ameliorated. Further research is needed to show the efficacy of the enzyme in diets with variable levels of inclusion of wheat and other high NSP containing cereals, as in the present study, only a single wheat level was used.

The exact mechanism underlying the observed effect of egg yolk color due to xylanase supplementation is not obvious and several pathways may be involved. It is known that carotenoids are absorbed alongside with lipids by passive diffusion [14]. However, prior the absorption step, the carotenoids need to be liberated from the feed matrix [14]. Thus, it can be hypothesized that due to the increased breakdown of NSPs by xylanase, the availability of carotenoids from the feed sources and consequently, those exogenously supplemented would be higher. Afterwards, carotenoids are emulsified together with lipids and form mixed micelles [14]. Egg yolk carotenoid content reflects the carotenoid content of birds’ diet, but it is also affected by other factors including the bird species, the bird health status, the efficiency of carotenoid absorption, the conversion of certain carotenoids to vitamin A and the feed matrix [46,47]. Usually, the diets used for laying hens are corn-soybean-based and their carotenoid content is low and therefore, exogenous pigments, such as lutein and canthaxanthin, are supplemented to the diet to improve yolk color [13]. It can be hypothesized that any improvement in the digestion and absorption of fatty acids by xylanase action also indirectly affected carotenoid absorption. Yolk carotenoids occur in a lipid-dissolved state and were possibly associated with large membrane lipoproteins [48]. The egg yolk fatty acid analysis showed that T4 treatment resulted in significant changes in egg yolk fatty acid profile. More specifically, polyunsaturated fatty acids, n-3 and n-6 fatty acid contents, increased significantly, while monounsaturated content reduced significantly in comparison with control diet (T1). A possible explanation of these findings is that the intestinal viscosity reduction caused by xylanase enzymes allowed for better availability of fats and other components. Moreover, xylanase enhances fat digestion by reducing bile salt deconjugation [12]. As a result, fat digestion and absorbance is favored by xylanase supplementation, which explains the changes in fatty acid profile and egg yolk coloration, as carotenoids are fat-soluble pigments.

Carotenoids are pigments that determine egg yolk color, as it is widely known that egg yolk color is directly related to the amount and types of carotenoids in layers’ diets. In this study, a significant increase of egg yolk canthaxanthin content was observed in dietary treatment T3 in comparison with T1 (control). This effect was measured after carotenoid extraction and quantification, while previous studies came in indirect conclusions about carotenoid content based on egg yolk coloration. Canthaxanthin is a red carotenoid that turns the typical yellow-orange color of the egg yolk to darker orange-red hue. The final color is determined by the relative contents of both yellow and red carotenoids [47]. More specifically, a yellow base is necessary to establish a good saturation, while red carotenoids act additively towards making the final color more orange-red [47]. Elsewhere, feeding of canthaxanthin for a week was enough for the pigmentation of egg yolk [49]. However, in the latter study, laying hens were fed a corn-based diet of approximately 60%, containing no wheat. In the current study, the main feed ingredient, wheat, has low contents of carotenoids compared to corn. Nevertheless, xylanase supplementation possibly enhanced canthaxanthin absorbance and bioavailability, while the rest of the individual carotenoids’ metabolism was not affected significantly by the enzyme supplementation. As aforementioned, the effect on canthaxanthin was significant in the T3 treatment. The description of the mechanism that defines the final egg yolk color confirms the higher score on egg yolk color in this experimental group.

To our knowledge, there is a lack of available literature about how dietary xylanase supplementation of laying hens may affect the fatty acid profile of egg yolk. An egg is one of the most widespread, low-cost functional foods. Producing eggs with an altered egg yolk fatty acid profile with lower costs could make such nutritive enriched eggs easily available to consumers. Consumer health is one of the main challenges in the food industry, hence these results are of great importance. Further research is proposed towards testing the efficacy of the enzyme in different types of feed ingredients.

## 5. Conclusions

Overall, the results of the present study show that in wheat-based diets in laying hens, xylanase supplementation could improve egg yolk color at all supplemented levels compared to the control diet. Meanwhile, the enzyme supplemented at the higher level (90,000 U/g) improved polyunsaturated and reduced monounsaturated egg yolk fatty acid content. It should be noted that for the purposes of the experiment, diets contained a high level of inclusion of wheat, which is not typical for laying hens’ diets. However, it can be suggested that xylanase enzyme should be supplemented in those diets containing high-NSP cereals in order to maintain (and improve) egg yolk color characteristics. Considering that nutritional value and organoleptic characteristics of eggs dictate consumers’ choices, producing eggs with an enhanced yolk fatty acid profile along with lower costs is likely to improve their marketability.

## Figures and Tables

**Table 1:** Main ingredients and nutrient analysis of the diets.

Ingredient (Labeling Information)	Diet (%)
Wheat soft	55.11
Soybean meal (47%CP)	15.40
Sunflower meal (34%CP)	10.00
Limestone	9.08
Corn, yellow lys	5.00
Soybean oil	3.73
Monocalcium phosphate	0.55
Mineral and vitamin premix ^a^	0.40
Sodium chloride	0.29
D,L-Methionine	0.16
L-Lysine HCl	0.15
Phytase	0.06
Sodium bicarbonate	0.05
Carophyll Red	0.04
L.Threonine	0.04
L-Valine	0.02
Total	100.00
**Calculated Nutrient Analysis**	**%AF**
Dry matter	89.00
Crude protein	17.50
Crude fat	4.99
Crude fiber	3.99
NSP	14.88
Starch	35.83
Ca	3.90
Phosphorus	0.53
Digestible phosphorus	0.40
Na	0.17
Cl	0.25
K	0.73
Lysine dig	0.76
Methionine dig	0.41
Cystine dig	0.26
Methionine + cystine dig	0.67
Triptophan dig	0.18
Threonine dig	0.53
Ile dig	0.61
Arginine dig	1.00
Valine dig	0.70
Metabolizable energy (ABE poultry based on CVB) Kcal/	2700.00

^a^ Provided per kg diet: Retinyl acetate: 4.2 mg; Cholecalciferol: 0.1 mg; α- tocopherol acetate: 31.25 mg; Menadione: 5.0 mg; Cyanocobalamin: 0.025 mg; folic acid: 1.0 mg; Choline chloride: 450 mg; Pantothenic acid: 12.5 mg; Riboflavin, 6.25 mg; Nicotinic acid: 43.75 mg; Thiamin: 3.0 mg; D-biotin: 0.1 mg; Pyridoxine: 5.0 mg; Manganese: 125 mg; Zinc: 112 mg; Iron: 62 mg; Copper: 10 mg; Iodine: 1.0 mg; Selenium: 0.15 mg.

**Table 2:** Substrate characterization in the control diet.

Ingredient	%
Moisture	9.61
Crude protein	18.04
Crude fat	5.63
Crude ash	13.50
Starch	35.24
Sugars	3.64
NDF	9.23
ADF	5.09
ADL	0.94
NSP	14.34
Hemicellulose	4.14
Cellulose	4.15

**Table 3:** Dosages and enzyme activities used in the present study.

Treatment	Dosage (g/t)	Intended Activity (U/kg)	Recovered Activity in Feed (U/kg)
T1 (control)	0	0	0
T2	10	30,000	35,861
T3	15	45,000	57,451
T4	30	90,000	111,936

**Table 4:** Treatment effects on egg yolk color (mean ± SD), measured visually with Yolk Color Fan^®^.

	T1	T2	T3	T4	*p*-Value
1st week	5.4 ± 0.58 ^a^	5.8 ± 0.86 ^ab^	7.4 ± 1.65 ^c^	6.7 ± 1.06 ^bc^	<0.001
4th week	5.6 ± 0.81 ^a^	7.1 ± 1.03 ^b^	7.4 ± 1.46 ^b^	7.6 ± 1.36 ^b^	<0.001
8th week	6.3 ± 1.08 ^a^	7.1 ± 1.29 ^ab^	8.3 ± 1.14 ^c^	7.8 ± 1.00 ^bc^	<0.001
12th week	5.7 ± 0.77 ^a^	6.6 ± 1.31 ^b^	7.7 ± 0.87 ^c^	6.9 ± 0.85 ^bc^	<0.001
Overall period	5.8 ± 0.87 ^a^	6.7 ± 1.23 ^b^	7.7 ± 1.34 ^c^	7.2 ± 1.15 ^c^	<0.001

T1: control-no enzyme; T2: 30,000 U/kg; T3: 45,000 U/kg; T4: 90,000 U/kg. Values are means ± SD. Values within the same raw with different superscripts are significantly different at *p* < 0.05.

**Table 5:** Treatment effects on egg yolk color (mean ± SD), measured instrumentally with Konica Minolta Chroma meter CR-410 using the L*a*b* color space.

	T1	T2	T3	T4	*p*-Value
L*					
4th week	74.5 ± 3.64 ^a^	73.7 ± 1.43 ^a^	73.8 ± 3.25 ^a^	80.0 ± 6.62 ^b^	<0.001
8th week	82.1 ± 2.85	82.9 ± 2.64	82.5 ± 2.25	83.4 ± 2.15	0.466
12th week	85.5 ± 1.29 ^a^	84.1 ± 1.82 ^b^	84.3 ± 1.07 ^ab^	84.5 ± 1.33 ^ab^	0.038
Overall period	80.7 ± 5.36	80.2 ± 5.11	80.2 ± 5.15	82.6 ± 4.46	0.057
a*					
4th week	3.3 ± 2.33 ^a^	4.1 ± 1.73 ^ab^	5.3 ± 1.96 ^b^	7.3 ± 1.70 ^c^	<0.001
8th week	4.0 ± 1.95 ^a^	6.7 ± 2.55 ^b^	8.5 ± 2.57 ^b^	7.4 ± 1.83 ^b^	<0.001
12th week	4.2 ± 1.80 ^a^	5.8 ± 3.38 ^ab^	8.2 ± 1.57 ^c^	6.7 ± 2.18 ^bc^	<0.001
Overall period	3.9 ± 2.03 ^a^	5.5 ± 2.80 ^b^	7.3 ± 2.51 ^c^	7.2 ± 1.90 ^c^	<0.001
b*					
4th week	35.5 ± 7.97 ^ab^	33.4 ± 5.10 ^a^	35.9 ± 8.65 ^ab^	41.5 ± 7.38 ^b^	0.020
8th week	39.0 ± 5.80 ^a^	44.7 ± 8.23 ^ab^	46.0 ± 5.88 ^b^	46.1 ± 7.70 ^b^	0.017
12th week	44.7 ± 3.47	47.5 ± 4.28	46.0 ± 3.52	46.9 ± 2.85	0.149
Overall period	39.8 ± 7.04 ^a^	41.9 ± 8.60 ^ab^	42.6 ± 7.88 ^ab^	44.8 ± 6.68 ^b^	0.014

T1: control-no enzyme; T2: 30,000 U/kg; T3: 45,000 U/kg; T4: 90,000 U/kg. Values are means ± SD. Values within the same raw with different superscripts are significantly different at *p* < 0.05.

**Table 6:** Egg yolk fatty acid profile at the end of the experimentation period.

Fatty Acid	Τ1	Τ2	Τ3	Τ4	*p*-Value
C14:0	0.29 ± 0.026	0.30 ± 0.030	0.29 ± 0.019	0.29 ± 0.026	0.679
C14:1n5	0.08 ± 0.017	0.08 ± 0.017	0.08 ± 0.014	0.08 ± 0.013	0.795
C16:0	25.72 ± 0.762	25.62 ± 1.051	25.36 ± 0.812	25.84 ± 0.969	0.369
C16:1n9	0.49 ± 0.082	0.47 ± 0.087	0.51 ± 0.099	0.45 ± 0.07	0.207
C16:1n7	3.65 ± 0.441	3.5 ± 0.713	3.53 ± 0.605	3.53 ± 0.584	0.823
C17:0	0.18 ± 0.02	0.19 ± 0.032	0.18 ± 0.03	0.18 ± 0.044	0.756
C17:1n7	0.11 ± 0.013	0.11 ± 0.025	0.11 ± 0.017	0.11 ± 0.009	0.377
C18:0	8.56 ± 0.585	8.71 ± 0.973	8.9 ± 0.891	8.74 ± 0.742	0.558
C18:1n9t	0.19 ± 0.02	0.18 ± 0.019	0.19 ± 0.021	0.18 ± 0.012	0.142
C18:1n9c	44.54 ± 1.988 ^a^	43.3 ± 2.581 ^ab^	43.74 ± 1.843 ^ab^	42.38 ± 2.096 ^b^	0.015
C18:1n7c	2.67 ± 0.243 ^a^	2.55 ± 0.27 ^a^	2.55 ± 0.181 ^a^	2.37 ± 0.181 ^b^	0.001
C18:2n6t	0.04 ± 0.012	0.04 ± 0.008	0.03 ± 0.006	0.04 ± 0.007	0.309
C18:2n6c	8.67 ± 1.513 ^a^	9.78 ± 1.927 ^ab^	9.58 ± 1.42 ^a^	10.9 ± 1.859 ^b^	0.001
C20:0	0.04 ± 0.007	0.04 ± 0.005	0.04 ± 0.007	0.04 ± 0.007	0.197
C18:3n6	0.1 ± 0.021	0.1 ± 0.022	0.1 ± 0.022	0.1 ± 0.027	0.627
C18:3n3	0.23 ± 0.052 ^a^	0.26 ± 0.066 ^a^	0.26 ± 0.056 ^a^	0.33 ± 0.081 ^b^	<0.001
C18:4n3	0.05 ± 0.014	0.05 ± 0.013	0.05 ± 0.015	0.06 ± 0.013	0.260
C20:1n9	0.29 ± 0.03	0.28 ± 0.03	0.28 ± 0.028	0.28 ± 0.03	0.248
C21:0	0.02 ± 0.005	0.02 ± 0.007	0.02 ± 0.006	0.02 ± 0.005	0.125
C20:2n6	0.11 ± 0.016 ^a^	0.13 ± 0.023 ^bc^	0.12 ± 0.016 ^ab^	0.14 ± 0.027 ^c^	<0.001
C22:0	0.07 ± 0.014	0.07 ± 0.012	0.08 ± 0.017	0.07 ± 0.012	0.174
C20:3n6	0.16 ± 0.02	0.16 ± 0.023	0.17 ± 0.062	0.16 ± 0.024	0.704
C20:4n6	1.88 ± 0.213 ^ab^	2.03 ± 0.251 ^bc^	2.05 ± 0.311 ^c^	1.86 ± 0.181 ^a^	0.022
C23:0	0.04 ± 0.007	0.04 ± 0.007	0.04 ± 0.012	0.04 ± 0.005	0.986
C20:5n3	0.02 ± 0.005	0.02 ± 0.007	0.02 ± 0.007	0.02 ± 0.005	0.643
C22:4n6	0.18 ± 0.027	0.2 ± 0.044	0.18 ± 0.03	0.19 ± 0.05	0.113
C22:5n6	0.86 ± 0.135	0.89 ± 0.167	0.77 ± 0.22	0.78 ± 0.144	0.062
C22:5n3	0.1 ± 0.022 ^a^	0.12 ± 0.031 ^ab^	0.11 ± 0.016 ^a^	0.13 ± 0.042 ^b^	0.025
C22:6n3	0.66 ± 0.106 ^a^	0.76 ± 0.126 ^b^	0.73 ± 0.127 ^ab^	0.72 ± 0.094 ^ab^	0.024

T1: control-no enzyme; T2: 30,000 U/kg; T3: 45,000 U/kg; T4: 90,000 U/kg. Values are means ± SD. Values within the same raw with different superscripts are significantly different at *p* < 0.05.

## Data Availability

The data presented in this study are available within the article.
